# Sex differences in lower limb neuromuscular function and asymmetry among 14–15 years old elite basketball players

**DOI:** 10.3389/fphys.2025.1704551

**Published:** 2025-11-18

**Authors:** Mengde Lyu, Evangelos Pappas, Ling Ding, Huaibin Qian, Jinglei Huang, Joshua Farragher, Charlotte Ganderton, Sophia Xenos, Jie Lyu, Jia Han

**Affiliations:** 1 Jiangwan Hospital of Hongkou District, Shanghai, China; 2 School of Health and Biomedical Sciences, RMIT University, Bundoora, VIC, Australia; 3 School of Physical Education, Shanghai University of Sport, Shanghai, China; 4 Graduate School of Shanghai University of Traditional Chinese Medicine, Shanghai, China; 5 College of Medical Instruments, Shanghai University of Medicine and Health Sciences, Shanghai, China; 6 College of Rehabilitation Sciences, Shanghai University of Medicine and Health Sciences, Shanghai, China

**Keywords:** basketball, asymmetry, adolecent, knee, lower limb

## Abstract

**Objectives:**

This study aimed to compare inter-limb asymmetry in balance, jumping and strength between male and female basketball players aged 14–15 years old.

**Design:**

Cross-sectional observational study.

**Methods:**

Sixteen male and 16 female youth elite basketball players performed the Y-Balance, two jump tests; the single-leg countermovement and broad jumps and isokinetic knee strength tests. The inter-limb asymmetry index was calculated.

**Results:**

The inter-limb difference in the Y-balance test and the single leg jumps was 5%–10%, while the difference in knee flexion and extension strength were 10%–35%. In terms of sex differences, the Y-balance asymmetry in males was significantly higher than that in females (males: 8.57% ± 4.01% vs. females: 4.04% ± 3.14%, *p* = 0.001), with no differences in any of the other outcomes. In terms of performance, female players performed significantly worse than their male counterparts in both types of single leg jumps and strength (*p* ≤ 0.01), except right knee extension (*p* = 0.07).

**Conclusion:**

In 14–15 years old basketball players, large side-to-side asymmetries exceeding 20% were observed, however, there were largely similar in males and females, suggesting that the higher risk of lower extremity injury in females may not be due to asymmetry. Although no lower limb injuries were present in these participants, inter-limb strength differences were nearly 20%, highlighting the need for specific inter-limb difference training. Longitudinal studies are warranted to explore whether this asymmetry is associated with a higher risk of injury in this group of basketball players.

## Introduction

Basketball is a high-intensity sport that involves frequent jumping, side-cutting, and changes of direction (Taylor et al., 2017), placing high demands on athletes’ strength, explosiveness, and balance (Ben Abdelkrim et al., 2007). To effectively execute rapid offensive and defensive transitions and complex skills in games, athletes require both well-developed physical fitness and balanced motor control between both sides of the body (Gonzalo-Skok et al., 2023; Wang et al., 2025a; Wang et al., 2025b). This bilateral symmetry is essential for maintaining high-level athletic performance and prevention for sports-related injuries (Cherni et al., 2021; Versic et al., 2021). In basketball, boys and girls exhibit distinct injury patterns; female players are more prone to knee injuries and concussions, with knee ligament sprains often requiring surgical intervention and prolonged absence from sports (Borowski et al., 2008). In contrast, male players more frequently sustain fractures and soft tissue contusions (Borowski et al., 2008). These sex-specific differences underscore the importance of developing targeted injury prevention strategies tailored to the unique training profiles of male and female athletes, thereby enhancing training effectiveness and ensuring athletic safety.

Epidemiological studies have demonstrated a marked increase in the incidence of anterior cruciate ligament (ACL) injuries among female athletes after puberty (Renstrom et al., 2008; Myer et al., 2013). Notably, sex differences in the number of ACL reconstructions increase during the second decade of life, with females having more ACL reconstructions in the 15–19 years age group (Renstrom et al., 2008). Adolescents typically undergo a rapid growth period after menarche (Cumming et al., 2017), during which the growth rate of bones significantly outpaces the development of muscle strength and neuromuscular control (Parry et al., 2024). This imbalance may contribute to asymmetrical development of strength and motor coordination between limbs in young athletes (Ding et al., 2024). Therefore, analyzing basketball players within this age range is critical for identifying early sex-related differences in neuromuscular development and inter-limb coordination, which may provide valuable insights for training optimization and performance monitoring during adolescence (Myer et al., 2013).

Inter-limb difference refers to differences between the left and right limbs of the body in terms of function, strength, and athletic performance, also known as asymmetry (Bishop et al., 2023). This difference is common among athletes, particularly those who engage in unilateral weight-bearing sports or specific movement patterns over a long period (Bishop et al., 2018). The leg dominance theory posits that athletes with greater functional asymmetries between their dominant and nondominant limbs may exhibit imbalances in neuromuscular control, movement coordination, and joint loading patterns, which could influence performance efficiency and motor control strategies (Pappas and Carpes, 2012; Weinhandl et al., 2017). Previous studies have used inter-limb asymmetry as an indicator for evaluating lower-limb function through tests such as the Y-Balance test, single-leg jump tests, and knee strength assessments (Smith et al., 2015; Olli et al., 2025; Pantazis et al., 2025). Asymmetries higher than 10%–15% are typically considered as exceeding the symmetry cutoffs and are used for predicting injury (Helme et al., 2021).

Studies indicated that males and females differ in pelvic structure, hip-knee control patterns, hormone levels, flexibility, and injury mechanisms (Mendiguchia et al., 2011; Hunter, 2014; Hunter and Senefeld, 2024). However, there is limited research systematically exploring the role of sex differences in inter-limb asymmetry (Guan et al., 2022; Ding et al., 2024), and whether these sex differences manifest during adolescence.

Considering the emergence of sex-specific knee injury patterns around puberty (Myer et al., 2013), there is an urgent need for standardized asymmetry calculation methods combined with multidimensional and commonly used functional assessments to systematically analyze lower-limb functional asymmetries in adolescent male and female basketball players. This new knowledge may lead to more targeted training and injury prevention strategies for youth athletes.

Accordingly, the aim of this study is to examine the inter-limb asymmetry index during knee strength and functional task performance in male and female adolescent basketball players. Our hypothesis is that female players will exhibit significantly greater inter-limb asymmetry index than male players.

## Methods

### Participants

A one-tailed independent samples t-test was planned to compare male and female participants. Given a large effect size observed in a pilot study (Cohen’s d > 1.0), a conservative expected effect size of d = 0.9 was used (Faul et al., 2007). With an alpha level of 0.05 and power of 0.80, the required sample size was estimated to be 32 participants (16 per group). Thirty-two youth basketball players (16 males and 16 females, age 14.47 ± 0.50 years, body mass 69.30 ± 18.08 kg, height 179.23 ± 11.22 cm, mean ± SD) were recruited in this study (Table 1). All participants were from the top eight teams in the country, comprising both male and female athletes who were elite youth basketball players at the national level in this age group. Each team had an average of six basketball training sessions and four strength and conditioning sessions per week. Considering that participants were adolescents, all informed consent forms were obtained from both participants and their parents. This study was approved by the Committee of the local University (approval number: 102772024RT021).

**TABLE 1:** Participant characteristics (mean ± SD).

Variables	Male (n = 16)	Female (n = 16)	Total (n = 32)
Age (y)	14.63 ± 0.48	14.19 ± 0.38	14.47 ± 0.50
Height (cm)	187.97 ± 8.49^a^	170.03 ± 5.95	179.23 ± 11.22
Body mass (kg)	80.36 ± 16.47^a^	60.06 ± 5.04	69.30 ± 18.08
Body fat (%)	13.96 ± 6.28^b^	18.15 ± 4.59	16.27 ± 6.10

^a^ significantly different with female (*p* < 0.001).

^b^ significantly different with female (*p* = 0.04).

#### Design

This study includes four lower limb tests: the Y-Balance test, single leg countermovement jump test, single leg broad jump and isokinetic knee strength test, with the order of tests randomized for each participant. Upon arriving at the testing site, participants first completed a standardized warm-up, which included jogging and dynamic stretching, followed by two practice jumps for each of the jump tests. After the 15-min warm-up, participants performed the four tests in a randomized order. A rest interval of 2 min was provided between repeated trials of the same test, and 5 min between different test types. To ensure consistency, the left side was tested first, followed by the right side.

#### Single leg countermovement jump test

The participants stood on one leg with their hands on their hips, then performed a countermovement to a self-selected depth and jumped as high as possible using the standing leg when ready. During the jump, additional swinging of the non-jumping leg was not permitted. The non-jumping leg was slightly bent at the knee, with the foot hovering near the ankle of the jumping leg (Madruga-Parera et al., 2020; Lyu et al., 2025). Three trials were conducted on the single-leg countermovement jump (SLCMJ) for each leg, with a 60-s rest between each trial. A trial was considered valid if the participant maintained balance on landing without touching the ground with the contralateral limb, performing additional hops. The jump test was Lirepeated if the jump movement was incorrect and repeated after adequate rest. The jumps were measured using the MYJUMP2 app, which has been proven to be a reliable jump measuring tool (Balsalobre-Fernández et al., 2015). The highest jump on each leg was used for subsequent data analysis (Madruga-Parera et al., 2020).

#### Single leg broad jump test

Participants stood on one leg at the 0 cm mark, performed a countermovement to a self-selected depth, and then jumped forward as far as possible while keeping their hands on their hips throughout the jump (Madruga-Parera et al., 2021). Participants were instructed to hold the landing for 2 s, and the distance was measured from the heel of the jumping foot (Madruga-Parera et al., 2021). Three trials were conducted on the single leg broad jump (SLBJ) for each leg, with a 60-s rest between each trial (Madruga-Parera et al., 2021). The trial was considered valid if the participant maintained balance on landing without touching the ground with the contralateral limb, performing additional hops, or exhibiting excessive trunk sway. The jump test was repeated if the jump movement was incorrect and repeated after adequate rest. The longest jump on each leg was used for data analysis (Madruga-Parera et al., 2021).

#### Y-balance test

The Y-Balance Test was conducted using the Y Balance Test Kit™ (Perform Better, West Warwick, RI). Participants placed their hands on their hips and stood on one foot at the standard position on the center axis of the testing board, ensuring that the second toe of the non-testing foot aligned with the red line in front. The non-testing foot was required to extend as far as possible to the anterior, posterior medial, and posterior lateral sides of the supporting leg, gently pushing the slider to its maximum distance without kicking it out. Finally, the non-testing foot was returned smoothly to the starting position without touching the ground or stepping on the slider for support (Overmoyer and Reiser, 2015). Any violations required a retest. Each participant performed three trials, with a 1-min rest between each trial. The composite score was calculated as follows: Composite Score = (Anterior + Posterior Medial + Posterior Lateral)/(3 × Length of Testing Leg) × 100. This composite score was used for statistical analysis (Overmoyer and Reiser, 2015).

#### Knee isokinetic strength test

Participants were seated with their hips flexed at 75° and secured to the dynamometer with Velcro straps around the thigh. Their upper bodies were stabilized using the dynamometer’s shoulder apparatus, and additional stabilization straps were used for the waist and distal femur. The knee joint axis was aligned with the dynamometer’s rotational axis at the lateral femoral condyle, leaving the leg unrestrained. A static gravitational correction was applied at a 30° knee flexion position to counteract the influence of gravity on the torque measurements. The range of movement was set from 10° (0° = full knee extension) to 90° and the angular velocity at 60°/s (Cinar-Medeni et al., 2015). The highest peak torque was normalized to body weight (PTs/kg) and it was used for the final data analysis (Ding et al., 2024).

### Statistical analysis

The descriptive data are presented as mean ± standard deviation (SD). Normality of the data distribution and homogeneity of variance were assessed using the Shapiro-Wilk and Levene methods, respectively. Inter-limb differences were calculated for all tasks using the following formula: (side with highest score)-(side with lowest score)/side with highest score * 100 (Bishop et al., 2023). Independent sample *t*-tests were used to compare the differences in performance for balance, jump and strength between males and females. The independent samples Mann-Whitney U test was used to assess asymmetry index differences between males and females. Cohen’ *d* effect sizes (ES) were computed for pairwise comparisons, defined as small (<0.2), moderate (0.2–0.5), and large (>0.8) based on the mean difference divided by the pooled standard deviation (Lyu et al., 2025). The Kappa coefficient was employed to assess the degree of agreement in asymmetry direction. The interpretation of Kappa values followed established thresholds: ≤0 indicating no agreement, 0.01–0.20 slight, 0.21–0.40 fair, 0.41–0.60 moderate, 0.61–0.80 substantial, and 0.81–0.99 denoting almost perfect agreement (Bishop et al., 2023). Statistical significance was set *a priori* at *p* < 0.05. All statistical analyses were conducted using IBM SPSS software version 26.0 (IBM, Armonk, New York, United States).

## Results

The sample consisted of 32 adolescents (16 males, 16 females) with a mean age of 14.5 years. Males were taller (mean 188.0 cm), heavier (mean 80.4 kg), and had lower body fat (mean 14.0%) than females (mean 170.0 cm, 60.1 kg, 18.2% body fat) (Table 1). Table 2 presents the performance in balance, jump, and strength of male and female players. Male players showed significantly higher jump and knee strength compared to females (*p* = 0.01, ES = 0.66–1.72), except for right leg knee extension (*p* = 0.07, ES = 0.47). There was no significant difference in balance ability between male and female players.

**TABLE 2:** The performance in balance, jump and strength of male and female youth players.

Test	Mean ± SD		ES (95% CI)	CV	*p*
	Male	Female		(%)	
Y- balance -L	0.88 ± 0.06	0.87 ± 0.05	0.18 (−0.47, 0.83)	2.3 (0.1, 4.5)	0.49
Y-balance -R	0.85 ± 0.08	0.87 ± 0.07	−0.28 (−0.93, 0.37)	2.6 (0.1, 7.9)	0.31
SLCMJ-L (cm)	17.95 ± 3.74	15.29 ± 1.97	0.89 (0.22, 1.55)	2.0 (0.3, 3.9)	<0.001
SLCMJ-R (cm)	17.88 ± 3.23	15.63 ± 2.10	0.82 (0.16, 1.48)	2.2 (0.2, 3.8)	<0.001
SLBJ-L (cm)	165.44 ± 21.45	141.13 ± 10.76	1.42 (0.71, 2.12)	1.8 (0.3, 3.3)	<0.001
SLBJ-R (cm)	165.31 ± 14.57	142.56 ± 13.28	1.72 (0.98, 2.45)	1.8 (0.3, 3.2)	<0.001
KF-L (N)	1.37 ± 0.35	1.15 ± 0.31	0.66 (0.01, 1.31)	4.7 (1.0, 7.7)	0.01
KF-R (N)	1.54 ± 0.32	1.25 ± 0.39	0.82 (0.16, 1.48)	6.5 (1.0, 9.9)	<0.001
KE-L (N)	2.46 ± 0.50	1.90 ± 0.44	1.20 (0.53, 1.86)	5.4 (1.0, 9.8)	<0.001
KE-R (N)	2.36 ± 0.50	2.09 ± 0.63	0.49 (−0.15, 1.14)	6.2 (2.0, 9.5)	0.07

Abbreviations: SLCMJ, single leg countermovement jump; SLBJ, single leg broad jump; KF, knee flexor; KE, knee extensor; L, left limb; R, right limb.

**FIGURE 1:**
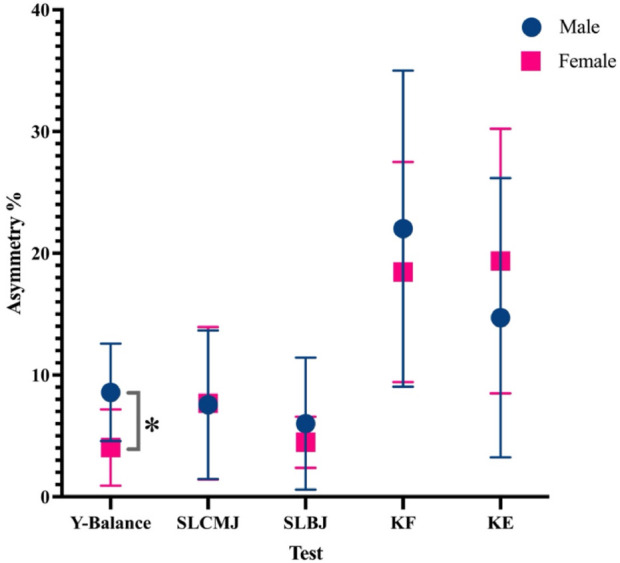
The difference in inter-limb asymmetry index between male and female players. Abbreviations: SLCMJ, single leg countermovement jump; SLBJ, single leg broad jump; KF, knee flexor; KE, knee extensor. * = significantly different (*p* = 0.001).

Figure 1 shows the difference in the inter-limb asymmetry index between male and female players. Only the asymmetry data for the Y-balance test were normally distributed. There is a significant difference in the asymmetry index of the Y-balance test (*p* < 0.01), with male athletes displaying significantly higher asymmetry than females, while there were no sex differences for any of the other tests.

**FIGURE 2:**
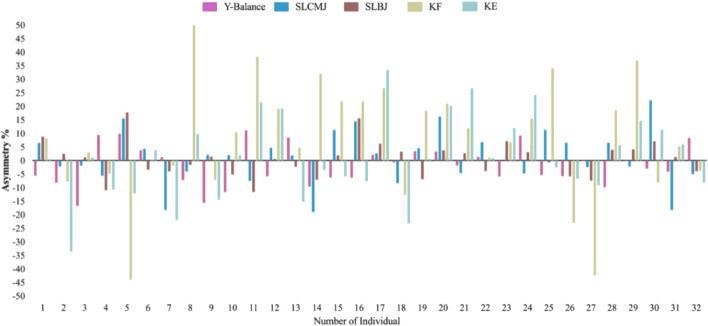
Individual inter-limb asymmetry data for all tests. Note: Above 0 means the asymmetry favors the left limb, and below 0 means the asymmetry favors the right limb. Abbreviations: SLCMJ, single leg countermovement jump; SLBJ, single leg broad jump; KF, knee flexor; KE, knee extensor.

Figure 2 shows the individual asymmetry score with direction. The consistency between tests was low (Kappa = −0.33–0.24), with only KF and KE showing a relatively higher level of agreement (Kappa = 0.61), indicating moderate consistency.

## Discussion

This study has established that no sex asymmetries exist for strength and jump performance in adolescent basketball players. The higher rate of knee injuries in female basketball players reported in the literature may be due to factors other than asymmetry. Though no sex differences were found, asymmetries were present within male and female data, including a 10%–35% difference in knee flexion and extension strength. Basketball is an asymmetrical sport, which may explain this finding, but also highlights the importance of further exploring the consequences of these asymmetries. In the only statistically significant sex finding, male players demonstrated higher balance asymmetry index than their female counterparts. As expected, males demonstrated higher jump and strength performance than females. The consistency analysis suggested that the direction of inter-limb asymmetry is highly task-specific and that different assessments may reflect distinct dimensions of lower-limb neuromuscular control.

Our findings indicated that, although female adolescent basketball players generally performed lower than males in terms of jumping ability and strength, they did not exhibit greater asymmetry in lower-limb functional tests compared to males. Specifically, there were no significant sex differences in asymmetry indices related to strength and explosive power, and females even showed significantly lower asymmetry in balance performance than males. This suggests that although inter-limb asymmetry may be related to sports injury risk, the higher incidence of lower limb injuries in female adolescent elite basketball players cannot be directly inferred to be caused by asymmetry based on our cross-sectional data. Considering of the asymmetry observed in all tests exceeded their respective CVs, indicating that the asymmetries can be considered as real. These findings challenge the commonly held assumption that greater inter-limb asymmetry contributes to the higher injury rates observed in female athletes. Instead, they suggest that other neuromuscular or biomechanical factors may play a more dominant role in injury susceptibility.

Male players exhibit significantly higher balance asymmetry indices than females, which may be attributed to sex differences in growth patterns and balance control abilities (Hewett et al., 2006; Hunter, 2014; López-Valenciano et al., 2019). Although males may develop greater muscle strength during puberty, unlike jumping and strength, balance relies more on nervous system coordination and fine motor control rather than muscle strength or body size differences (Han et al., 2015). Previous studies have reported sex differences in lower limb proprioception, as proprioception is fundamental to balance control, asymmetries in balance may reflect proprioceptive asymmetries (Han et al., 2013; Lee et al., 2015). At the same time, this suggests that sex could be a factor influencing asymmetry, and future research should further analyze the impact of sex on asymmetry. These results can guide practitioners in designing targeted injury prevention and training programs for athletes of different sexes to effectively promote balance in lower limb performance. Future research should incorporate functional assessments or performance-based measures to further determine whether such differences in asymmetry have meaningful practical consequences.

Previous studies have raised doubts about using the inter-limb asymmetry index as a return-to-sport (RTS) cutoff. For example, some research has shown that among healthy adolescent athletes, only 45% meet the criteria for all jump tests (asymmetry index ≤10%) (Magill et al., 2021). Additionally, other studies have indicated that directly applying adult standards to younger participants is inappropriate (Pauw et al., 2023). Therefore, research suggests that the optimal inter-limb asymmetry index remains undetermined and may vary depending on the type and level of sport activity in which an individual engages. Researchers have suggested using comparisons to normative values instead of the limb symmetry index as it may be a more sensitive measure (Gokeler et al., 2017). Given the potential presence of bilateral deficits, caution should be exercised when using inter-limb asymmetry index (Dingenen and Gokeler, 2017). Based on our results, each test should have independent criteria to serve as reference standards for RTS decisions of adolescent (14–15 years old) basketball players. Although Thomas et al. reported knee flexor and extensor asymmetry indices of 13.11 ± 10.10 and 11.98 ± 11.18, respectively, in 17 basketball players, their participants were generally 3–4 years older than those in the present study which were in the midst of pubertal development (Thomas et al., 2017). Additionally, the large standard deviations reported (similar to those in our findings) should be taken into consideration (Thomas et al., 2017). Therefore, a threshold of 10%–15% may be appropriate for jump and balance tests among adolescent basketball players; however, asymmetry levels observed in the isokinetic strength test of the knee clearly exceeded this range. This may be because jump and balance tests are more complex compared to knee strength tests, demanding higher neuromuscular processing due to the coordination of multiple joints and muscle groups (Place and Millet, 2020). Consequently, the asymmetry index for jumping and balance tests may not reflect the precise asymmetry of a single joint, as they represent the combined functioning of multiple joints. This suggests that practitioners may assign different asymmetry thresholds for different tests (Bishop, 2021), and it is necessary to analyze the strength asymmetry of each joint, as this can provide more information to help players achieve balanced development of bilateral limb capabilities, especially for sports like basketball that require well-developed abilities in both limbs (Afonso et al., 2022). Additionally, consistent monitoring of players’ inter-limb asymmetry throughout long-term training is recommended, as this could provide valuable reference data for RTS decision-making in case the athlete may have an injury in the future.

### Study limitations

This study also has some limitations. First, although the players all come from the same region and undergo very similar training, the sample size is still small, and the findings may not extend to players with different training volumes or talent pathways. Second, the participants in this study were only aged 14–15 years. Future research should include participants of a wider age range to provide more comprehensive information.

## Conclusion

Our results indicate that male adolescent elite basketball players exhibit significantly higher jump and strength performance, but also a higher balance asymmetry index, compared to females. Although the study population did not have lower limb injuries, the inter-limb difference in lower limb strength reached approximately 20%. These findings suggest that specific inter-limb difference training may needed to address this significant asymmetry, and a longitudinal study is warranted to explore whether this asymmetry is associated with a higher risk of injury in this group of basketball players.

## Data Availability

The raw data supporting the conclusions of this article will be made available by the authors, without undue reservation.
